# Monoclonal antibodies for prophylaxis and therapy of respiratory syncytial virus, SARS-CoV-2, human immunodeficiency virus, rabies and bacterial infections: an update from the World Association of Infectious Diseases and Immunological Disorders and the Italian Society of Antinfective Therapy

**DOI:** 10.3389/fimmu.2023.1162342

**Published:** 2023-05-15

**Authors:** Susanna Esposito, Gayatri Amirthalingam, Matteo Bassetti, Francesco Blasi, Francesco Giuseppe De Rosa, Natasha B. Halasa, Ivan Hung, Albert Osterhaus, Tina Tan, Juan Pablo Torres, Antonio Vena, Nicola Principi

**Affiliations:** ^1^ Pediatric Clinic, Pietro Barilla Children’s Hospital, Department of Medicine and Surgery, University of Parma, Parma, Italy; ^2^ Immunisation and Countermeasures Division, National Infection Service, Public Health England, London, United Kingdom; ^3^ Division of Infectious Diseases, Department of Health Sciences (DISSAL), University of Genova, Genoa, Italy; ^4^ IRCCS Ospedale Policlinico San Martino, Genoa, Italy; ^5^ Department of Pathophysiology and Transplantation, Università degli Studi di Milano, Milan, Italy; ^6^ Respiratory Unit and Cystic Fibrosis Center, Fondazione IRCCS Cà Granda Ospedale Maggiore Policlinico Milano, Milan, Italy; ^7^ Department of Medical Sciences, Infectious Diseases, University of Turin, Turin, Italy; ^8^ Department of Pediatrics, Vanderbilt University Medical Center, Nashville, TN, United States; ^9^ Department of Medicine, School of Clinical Medicine, Li Ka Shing Faculty of Medicine, The University of Hong Kong, Pokfulam, Hong Kong SAR, China; ^10^ Department of Infectious Disease and Microbiology, The University of Hong Kong-Shenzhen Hospital, Shenzhen, China; ^11^ Research Center for Emerging Infections and Zoonoses, University of Veterinary Medicine Foundation, Hannover, Germany; ^12^ Division of Infectious Diseases, Feinberg School of Medicine of Northwestern University, Chicago, IL, United States; ^13^ Department of Pediatrics and Pediatric Surgery, Facultad de Medicina, University of Chile, Santiago, Chile; ^14^ Instituto Sistemas Complejos de Ingeniería (ISCI), Santiago, Chile; ^15^ Università degli Studi di Milano, Milan, Italy

**Keywords:** bacterial infection, COVID-19, HIV, infectious diseases, monoclonal antibodies, rabies, RSV

## Abstract

Monoclonal antibodies (mABs) are safe and effective proteins produced in laboratory that may be used to target a single epitope of a highly conserved protein of a virus or a bacterial pathogen. For this purpose, the epitope is selected among those that play the major role as targets for prevention of infection or tissue damage. In this paper, characteristics of the most important mABs that have been licensed and used or are in advanced stages of development for use in prophylaxis and therapy of infectious diseases are discussed. We showed that a great number of mABs effective against virus or bacterial infections have been developed, although only in a small number of cases these are licensed for use in clinical practice and have reached the market. Although some examples of therapeutic efficacy have been shown, not unlike more traditional antiviral or antibacterial treatments, their efficacy is significantly greater in prophylaxis or early post-exposure treatment. Although in many cases the use of vaccines is more effective and cost-effective than that of mABs, for many infectious diseases no vaccines have yet been developed and licensed. Furthermore, in emergency situations, like in epidemics or pandemics, the availability of mABs can be an attractive adjunct to our armament to reduce the impact. Finally, the availability of mABs against bacteria can be an important alternative, when multidrug-resistant strains are involved.

## 1 Introduction

Passive immunization through the administration of serum from previously infected or immunized human donors or animals, is known for many years as an effective measure to prevent and treat several infectious diseases. The first example in this regard dates back to the end of the 19^th^ century when anti-diphtheria serum was used to treat and heal several hundred children with this disease (1). Since then, passive immunotherapy with convalescent human serum was used for prophylaxis and therapy of several viral diseases such as measles, varicella-zoster, the 1918 influenza pandemic and Ebola fever (2). Due to logistic problems and occasional severe side effects, serum therapy was progressively abandoned, particularly when, after the second world war, technology improvements allowed the preparation of pooled human immunoglobulin. This could be administered intramuscularly and intravenously and was shown to be significantly more effective and better tolerated (3). Several of these preparations are still on the market and used to treat viral diseases, including hyperimmune preparations against rabies virus, cytomegalovirus, hepatitis B and C viruses, vaccinia virus, varicella-zoster virus, respiratory syncytial virus (RSV) and West Nile virus (4).

However, serum-derived immunoglobulin G (IgG) preparations have their limitations. These preparations contain IgGs directed to all the epitopes of the agent that had previously infected the donor. This means that they contain a large and diverse population of antibodies, most of which are not neutralizing the agent concerned. Moreover, serum-derived IgG preparations harbor the risk of pathogen transmission and significant batch-to-batch variation in antibody content. Finally, obtaining immune donors can be difficult, especially at the start of an epidemic or pandemic. To overcome these limitations, the development of monoclonal antibodies was originally pioneered by Nobel laureates Köhler and Millstein (5). Afterwards, monoclonal antibodies (mABs) and their therapeutic use, especially in cancer, immunological and infectious disease therapy, has really taken of (6, 7). Despite the pipeline needed to produce mABs at a quality that is suitable for human use is complex and expensive, therapies using mABs can be significantly more effective and safer than those using conventional human IgGs. They usually target a single epitope of a preferably highly conserved antigen of a virus or a bacterial pathogen. The target epitope is chosen among those that play the major role in conditioning the development of an infection or a tissue damage. Consequently, mABs are more specific and, consequently, more potent. The risk that this specificity results in increased antigenic escape in infection due to mutation by infectious agents can be overcome with the use of mixtures of two or more mAbs specific for distinct protective epitopes or of bi-specific mABs (8). Moreover, a mAB can be engineered with a significant prolongation of its elimination half-life. This extends long-term clinical effects, favoring administration and duration of clinical efficacy. Finally, they have reduced the risk of side effects, like serum sickness and anaphylaxis that for instance can occur with animal-derived polyclonal preparations (9, 10).

In this paper, characteristics of the most important mAB that have been already licensed or are in advanced development for use in prophylaxis and therapy of infectious diseases will be discussed.

An in-depth research and review of the medical literature was performed. The MEDLINE–PubMed database was searched from January 1, 2000 to December 31, 2022 to collect the literature. The search included randomized placebo-controlled trials, controlled clinical trials, double-blind, randomized controlled studies, and systematic reviews of the last 10 years. The following combinations of keywords were used: “RSV” OR “SARS-CoV-2” OR “COVID-10” OR “HIV” OR “rabies” OR “bacteria” AND “prevention” AND/OR “treatment” AND/OR “vaccine” AND/OR “antibody” AND/OR “monoclonal antibody”. We also performed a manual search of the reference lists of the obtained studies. The search was limited to English-language journals and full papers only.

## 2 Monoclonal antibodies against viruses

### 2.1 Respiratory syncytial virus

RSV is a common respiratory virus that primarily circulates during fall, winter, and spring and usually causes only mild to moderate cold-like symptoms in healthy older children, adolescents, and adults (11). On the contrary, it can be very dangerous in infants and older adults in whom it may cause very severe bronchiolitis and pneumonia leading to immediate and long-term dramatic medical, social and economic consequences (12–14). It has been calculated that in the USA, before the COVID-19 pandemic, every year RSV caused in children <5 years of age 2.1 million outpatient visits, 58 000 hospitalizations and 100-300 deaths (15). Preterm infants, those with congenital heart or chronic lung disease neuromuscular disorders or Down’s syndrome are those at the highest risk. Among adults 65 years RSV was considered responsible of 177,000 hospitalizations and 14,000 deaths, mainly in those with severe underlying disease (15).

Epidemiology of RSV infection has significantly changed during the first year of the COVID-19 pandemic, with a substantial reduction of the total number of severe RSV infections, probably due to the impact of the non-pharmaceutical measures put in place by health authorities to reduce COVID-19 circulation (16, 17). However, a significant increase in RSV cases has been already evidenced in 2021 when restrictions were partially or totally withdrawn. This implies that the epidemiology of RSV will soon return to the same or perhaps even more severe, than what was experienced before the pandemic. The reduced circulation of the virus for a couple of seasons may have increased the number of susceptible subjects, thus increasing the risk of a greater number of infections in the fall and winter seasons in moderate climate zones (18).

To reduce RSV disease burden in infants and the elderly, repeated attempts to develop vaccines and mABs were made. Vaccine development was initiated in the 1960s, but it was strongly delayed after a catastrophic failure of the first preparation that had led to the death of two infants (19). Only recently effective and safe preparations have been produced. Several vaccines are in advanced phase of development, and it seems likely that in the next few years several preparations will be approved, that can prevent or mitigate RSV diseases in patients at risk (20, 21). Fortunately, effective and safe mABs, palivizumab (PV) and motavizumab (Mo; MEDI-524, Numax) were developed. MO was proven to be effective for treatment of RSV in infants, including those in higher risk groups, but after the first studies it was no longer developed by the manufacturing company. In contrast, PV has been largely studied in both adults and children. It is not recommended for use in adults, despite some authors suggested that it may represent a safe and effective measure for prevention of RSV disease also in this group of subjects (22). On the contrary, it is not only authorized for use in selected group of children, but it represents the only present measure capable of significantly reduce the total burden of RSV in this pediatric population.

#### 2.1.1 Palivizumab

Palivizumab (PV) is a humanized mAB produced by recombinant DNA technology, targeting a highly conserved region on the extracellular domain of mature RSV F protein, referred to as antigenic site II or site A (23). PV exhibits neutralizing and fusion-inhibitory activity against the virus, so impairing its replication and spread. Due to the evidence that PV could be 45%–82% effective against RSV-related hospitalizations in high-risk infants without risk of severe adverse events (23–25), this mAB was initially authorized for use in preterm babies and in several groups of neonates and infants suffering from underlying disease potentially associated with greater susceptibility to RSV infection or more severe RSV disease (26). Over the years, these indications have been modified several times according to the evidence that the global benefits of PV prophylaxis were lower than expected. Effect on RSV hospitalizations was poor, impact on mortality was not measurable, and effect on the development of subsequent wheezing was minimal (27–29). Failures were mainly ascribed to the presence of RSV strains with mutations in the target antigenic site of the F protein (30). Groups of children for whom PV prophylaxis was considered recommendable was progressively reduced.

Presently, PV is authorized for prevention of RSV disease in infants: 1) who are born < 29 weeks’ gestation and are <12 months old at the start of the RSV season; 2) who develop chronic lung disease of prematurity, defined as gestational age <32 weeks and a requirement for >21% oxygen for at least the first 28 days after birth; 3) who are <12 months old and suffer from a hemodynamically significant congenital heart defect, including those with acyanotic heart disease that needs medication to control congestive heart failure and requires cardiac surgical procedures and those with moderate to severe pulmonary hypertension. Moreover, selected cases of children with anatomic pulmonary abnormalities or neuromuscular disorder and severe immune deficit may be considered for PV prophylaxis (31).

The approved dose of palivizumab is 15 mg/kg of body weight, administered intramuscularly once a month for a maximum of 5 months, just the duration of the RSV season that usually occurs from November to March in the Northern hemisphere. As the drug has an elimination half-life in pediatric patients varying from 17 days (32) to 26.8 days (33), this schedule of administration assures persistent PV serum concentrations higher than the minimum protective level of 40 μg/mL (33, 34). Compliance to the suggested scheme of administration is critical to maintain the maximum prophylaxis efficacy. The first dose should be given before the start of the RSV season. Earlier administration leaves the infant exposed to infection in the last months of RSV season. On the contrary, the opposite occurs if the administration is late compared to the start of the RSV season. Unfortunately, compliance was frequently found suboptimal with rates lower than 50%. Higher values were found only when prophylaxis was given through monthly home visits by a health professional or reminder telephone calls to parents or caregivers and extensive counseling of parents were planned (35).

Finally, PV is very expensive, and its cost/effectiveness ratio is debated. A systematic review of the studies published until 2018 showed that from a payer perspective, PV was relatively cost-effective in infants with bronchopulmonary dysplasia, congenital heart disease, term infants from specific remote communities, and preterm infants with and without lung complications (36). Economic analyses have failed to demonstrate overall savings in health care dollars because of the high cost if all infants who are at risk receive prophylaxis. This finding, together with the intricacy of the intramuscular administration has led to several attempts to find easier and less expensive PV administration. The intranasal use of PV and its substitution with a biosimilar have been proposed (37). Targeted localized use of prophylactic and therapeutic antibodies is suggested as a potential solution to reduce expenses as they can be produced without the stringent regulatory requirements of manufacturing injectable antibodies and lower doses are needed when the infection is restricted locally to an external surface (37). A trial enrolling both adults and preterm infants given PV by intranasal route, but no results have been till now published (38). A significant reduction of expenses can also derive from the production and use of a PV biosimilar, particularly when, as in the case of PV, the patent on the technology of the original antibody is expired and its use is strongly recommended at least for a group of subjects. A PV biosimilar is in preclinical development in Netherlands and Spain, but even in this case there aren’t reliable data regarding results of the human challenge (39). It is obvious that a PV biosimilar nasally administered would solve at least two of the problems limiting the extensive use of PV, i.e. parenteral administration and high cost. However, the problem of repeated administration would remain for all the months of the RSV season, which could create organizational problems that strongly limit the achievement of high levels of coverage, especially in countries with a health system of limited efficiency.

#### 2.1.2 New monoclonal antibodies effective against respiratory syncytial virus (RSV)

In recent years, significant advances in the knowledge of RSV fusion protein structure, antigenicity, and immunogenicity have led to the development of new mABs with greater efficacy and fewer logistical barrier to administration than PV. To increase efficacy, mABs targeting highly neutralization-sensitive epitopes sited on the pre-fusion F protein where produced (40). Moreover, to make administration easier, mABs were engineered with multiple substitutions, generally the M252Y/S254T/T256E (YTE) mutation, within their Fc region. This was associated with a considerable prolongation of the antibody elimination rate with the consequent possibility of obtaining, even with a single administration, protective concentrations against RSV for a longer period, corresponding to that of the entire RSV season (41).

Among all the possible new anti RSV mABs, the one with the most advanced development is nirsevimab (NSM), that possesses all the innovative structural characteristics cited above to improve efficacy and make administration easier. For this mAB, a large series of pharmacokinetic and clinical findings indicate that a single administration of NSM at the recommended dosage can significantly reduce in all the children, regardless gestational age and underlying disease, the risk of RSV-induced LRTI and related hospitalization throughout the season in which RSV circulates (42). Starting from these premises, this mAB was approved in Europe (43). Later will be the introduction of NSM to the market in the USA where the request for authorization of use is delayed compared to Europe.

Initially, efficacy and safety of NSM was tested in preterm infants in a randomized, placebo controlled clinical trial enrolling 969 preterm (29 weeks 0 days to 34 weeks 6 days of gestation) infants and 484 matched controls (44). Participants received NSM, at a dose of 50 mg in a single intramuscular injection, or placebo at the start of an RSV season. Throughout the 150-day period after the dose, the incidence of RSV-associated medically attended lower respiratory tract infections (MALRTIs) was significantly lower (70.1%) in treated children than in controls. RSV disease was diagnosed in 2.6% vs 9.5% of the children, respectively (P<0.001). Even better were the results of prophylaxis administration when hospitalization rate due to RSV-associated MALRTIs was considered. In this case, reduction in treated infants was 78.4% lower (95% confidence interval [CI], 51.9 to 90.3). Only 0.8% of children receiving NSM were hospitalized compared to 4.1% of those given placebo (P<0.001). Safety of NSM was considered to be good as incidence of adverse events, including those severe and high, was quite similar in treated infants and controls and considered unrelated to the mAB administration and probably associated with prematurity. No notable hypersensitivity reactions occurred. The analysis of pharmacokinetic data collected in children given NSM clearly explained why 50 mg were adequate to assure a long-term effect. Serum elimination half-life was estimated to be 62.5–72.9 days. Moreover, on day 151 after administration, serum concentrations in about 98% of NSM recipients were above the 90% effective concentration level of 6.8 μg/mL (44).

Later, NSM was tested in late preterm and term infants in a randomized, placebo-controlled phase 3 trial enrolling a total of 1,478 children, among whom 987 received NSM (50 mg if they weighed <5 kg or 100 mg if they weighed ≥5 kg) and 491 were treated with placebo (45). Results were quite like those previously reported in preterm infants as efficacy of NSM in the prevention of RSV-associated MALRTIs was 74.5% (95% CI, 49.6 to 87; P<0.0019). Only 1.2% of children who received NSM prophylaxis suffered from an RSV-associated MALRTIs compared to 5.0% of those receiving placebo. Reduction of hospitalizations was also relevant as hospitalization rates due to RSV were 0.6% in the NSM group and 1.6% in the placebo group. Efficacy of NSM was 62.1% (95% CI, −8.6 to 86.8). Unfortunately, probably due to the low number of hospitalized children in both groups the difference in hospitalization was not statistically significant (P=0.07l). Moreover, subgroup analysis revealed that relative risk reduction of medical-attended RSV-associated LRTI was age-related as it was higher in children aged > 3 months at randomization (92.2% vs 58.8%) and in those weighting ≥ 5 kg at day 1 of the study (85.7% vs 52.4%) (45). Pharmacokinetic data showed that serum concentrations of NSM associated with protection could be detected through 150 days after administration across age and weight subgroups (46). NSM was safe and well tolerated. The total number of adverse events was similar in treated children and controls (13.4% vs 12.8%) as was the incidence of severe adverse events (6.8% vs 7.3%), none of which were considered related to NSM nirsevimab or placebo (46).

These findings, together with relatively low price, made several experts think that a universal use of RSV prophylaxis in children was possible (47–49). Instead of a few, highly selected, children, these new mABs could have allowed the protection from RSV disease and related problems all the infants and toddlers. Recently collected data seem to confirm experts’ expectations. A static decision-analytic model of the US birth cohort during its first RSV season has estimated NSM impact on RSV-disease and related costs (50). Assuming a 71% and 80% uptake rates in healthy infants and palivizumab-eligible infants, respectively, together with an immediate onset and a 5-month duration of protection, it has been calculated that using NSV 290 174 RSV-medically attended lower respiratory tract illness and 24 986 hospitalizations could be avoided and $612 million 2021 USD saved (50). Similar findings were reported by Voirin et al. who developed a dynamic mathematical model capable of providing initial insights into the direct and indirect effects of NMV on RSV transmission (51). Assuming a 71% coverage and 70% efficacy, these authors reported that administering NMV to all the infants entering their first RSV epidemic season or born during the epidemic season (1 November–31 March), a 50% and 35% reduction of MALRTIs among infants aged 0–6 months and 6–12 months during the RSV epidemic season could be obtained, respectively, independently of any effect of the mAB on viral shedding. Moreover, if it is assumed that NMV administration could reduce viral shedding with about 50%, a further 16% increase of avoided MALRTIs could be calculated (51).

Clesrovimab (formerly MK-1654) is a mAB similar to NSM as it targets the site IV of the RSV pre-fusion F protein and the same YTE mutation (52). Studies are ongoing to evaluate whether clesrovimab would reduce the incidence of RSV-associated MALRTI from Days 1 through 150 postdose compared to placebo in presence of an appropriate safety profile.

### 2.2 SARS-CoV-2

Through March 21, 2023, a total of 761,071,826 COVID-19 cases have been reported to the WHO, with 6,879,677 deaths (53). These numbers largely underreport the true burden of SARS-CoV-2 infection, as a great number of cases occurring in healthy subjects remained asymptomatic, despite significantly contributing to the circulation of the virus and the development of new COVID-19 cases among the susceptible population (54). The impact of COVID-19 was dramatic not only for the health system, but also from a social and economic point of view (55). Most of the severe cases were diagnosed in the elderly and in people with underlying chronic severe disease regardless of age, although severe COVID-19 requiring hospitalization and leading to death have been repeatedly reported even in the healthy adult population and relevant social problems were evidenced in adolescents and young adults (56). Children, especially the youngest, were initially marginally involved but, when a significant number of adults became protected by previous infection or vaccine immunization, prevalence of pediatric COVID-19 cases significantly increased (57). The emergence of variants against which children were unprotected further increased the percentage of children found positive to SARS-CoV-2 infection. In the period October 10, 2021, to September 29, 2022, when the Omicron variant became progressively dominant, the percentage of pediatric COVID-19 cases on the total number of COVID-19 cases diagnosed in the USA rose from 16.6% to 18.4% (58). Moreover, clinically relevant long-term consequences of COVID-19, including the multisystem inflammatory -64syndrome of children (MIS-C) (59) and neonates (MIS-N) (60) and long-COVID (61–63) were repeatedly described.

To prevent and treat COVID-19, together with antivirals and vaccines, development of mABs was planned. The evidence that the SARS-CoV-2 spike protein was critical for SARS-CoV-2 infection and COVID-19 development led to the conclusion that inactivation of S protein functions by specifically prepared mABs could be an effective measure (64). Development of mABs against S protein was strongly accelerated and in the first months after pandemic declaration several preparations became available (65).

Most of mABs targeted epitopes on the receptor binding domain (RBD) contained in the subunit 1 of the S protein that allows SARS-CoV-2 to attach to its receptor, the angiotensin-converting enzyme 2 (ACE2) on the host cell. A small number of mABs was directed against other components of the S1 subunit such as the N-terminal domain (NTD) and the receptor-binding motif (RBM) (66). After careful evaluation and the evidence that they could positively influence the course of COVID-19 infection without significant risk of adverse events even in the pediatric population (67–69), most of them were licensed for use, alone or as combinations. Unfortunately, the anticipated efficacy of the different mABs varied dramatically depending on the circulating virus variant. Several of them (sotrovimab, bamlanivimab plus etesevimab, casirivimab plus imdevimab), initially highly effective against the original SARS-CoV-2 variants of concern (VOCs), remained only partially effective against the Delta variant and lost any effect when the Omicron variant, particularly the most recent BA.4 and BA.5 subvariants, became predominant. Presently, only bebtelovimab (BEB) (70–72) and the combination tixagevimab-cilgavimab (TC) (73–75) retain *in vitro* neutralization activity against at least some of the circulating Omicron subvariants. In particular, BEB is considered potentially *in vivo* active against Omicron BA.5 and Omicron BA.4.6/BF.7 and TC only against BA.5. Both remain authorized for emergency use in both adults and children (aged > 12 years and weighting >40 kg). BEB is authorized for patients with mild to moderate COVID-19 who are at high risk for progression to severe COVID-19 and cannot use the antivirals oral paxlovid or intravenous remdesivir (76). However, lacking data showing that BEB can be effective in patients with severe COVID-19, this mAB is not authorized for use in hospitalized patients. Moreover, it cannot be ignored that the true efficacy of BEB should still be carefully monitored as the emergency authorization has been decided, although studies carried out in patients infected by the Omicron variant were lacking and only *in vitro* tests showing Omicron variant inactivation were available. The data supporting BEB authorization were collected in a group of 714 patients with mild to moderate COVID-19 enrolled in a period that Alpha and Delta variants were predominant (77). Compared to placebo, BEB led to a reduction in viral load on Day 5 after treatment, in time to sustained symptom resolution and in rates of COVID-19 related hospitalization and death through Day 29 (77). Moreover, no data have been collected in children and the authorization for use in those aged > 12 years and weighting > 40 kg was decided considering the similarity between these patients and adults in COVID-19 course and response to drug administration.

The combination tixagevimab/cilgavimab is authorized for emergency use for pre-exposure prophylaxis against COVID-19 for immunocompromised individuals or those who cannot be vaccinated or mount a satisfactory post-vaccination immune response (78). Also in this case, results of the study showing the positive effect of the combination were collected before the emergence of the Omicron and deserve confirmation (79). Pediatric studies are still lacking.

### 2.3 Examples of other viruses

#### 2.3.1 Human immunodeficiency virus

Antiviral therapy has significantly improved the prognosis of patients with human immunodeficiency virus (HIV) infection. However, in some cases, despite complex antiviral therapy, multidrug resistant HIV strains emerge with increased risk of severe AIDS development and death. To face these problems antibody-based strategies were considered and some mAbs were developed (80). Ibalizumab is the most largely studied. It is a recombinant humanized immunoglobulin (Ig) G4 mAB derived from mouse and acts inhibiting HIV entry into the CD4 T cell. The mAB binds to the CD4 T cell extracellular domain 1 and 2 so preventing those conformational changes within the complex of the CD4 T cell and the HIV envelope gp120 that allow viral fusion and cell entry (81) Ibalizumab is approved for intravenous use as part of a combination antiretroviral regimen in heavily treatment-experienced patients with multidrug resistant (MDR) HIV-1 infection who did not respond to the current antiretroviral regimen (82). This mAB was approved after a study, including 40 patients with limited treatment options, had shown that addition of this mAB to their failing antiretroviral regimen could lead to a very fast reduction of HIV-RNA levels and after 24 weeks of treatment 43% of participants achieved HIV RNA suppression (83).

#### 2.3.2 Rabies

Rabies is a fatal, acute and progressive encephalomyelitis that is estimated to cause about 60 000 human deaths each year. Despite it is an ancient illness for which the first vaccine was developed by Louis Pasteur more than 130 years ago, it is still considered one of the most neglected diseases. No effective treatment is presently available and research in this regard is very poor. Only in recent years some improvement has been made as far as prevention is concerned (84).

Rabies is caused by neurotropic viruses from the *Rhabdoviridae* family belonging to the Lyssavirus genus. Virus genome encodes five proteins: nucleoprotein (N), phosphoprotein (P), matrix protein (M), glycoprotein (G) and the RNA polymerase (L). G protein is sited on the surface of the virus envelope and is the main target of the immune response. Five antigenic sites within G protein have been identified. Among them, sites I and III are considered the most important as neutralizing antibodies from human vaccines primarily act against them (85).

Rabies can be prevented with post-exposure prophylaxis, composed of vaccines and anti-rabies immunoglobulins; the vaccine alone is not enough as, in some cases, the disease develops before the vaccine can take effect. Prophylaxis should be administered as fast as possible and no later than 7 days from exposure. It should be considered in patients with category II lesions (nibbling of uncovered skin, minor scratches or abrasions without bleeding) and is mandatory in patients with category III lesions (single or multiple transdermal bites or scratches, contamination of mucous membrane or broken skin with saliva from animal licks, exposures due to direct contact with bats) (86). Immunoglobulins derived from the blood plasma of horses or humans commonly used for post-exposure prophylaxis of rabies have several limitations relating to supply, cost, and quality (87). To overcome these limits, several mABs have been developed, and five have reached clinical trials. Rabishield and the combination miromavimab plus docaravimab are the preparations in the most advanced stage of development. They are licensed in India for post-exposure prophylaxis in conjunction with vaccine administration (88). Rabishield acts binding to a conformational epitope of the rabies G protein and, due to this mono-specificity, has two potential limitations, lack of neutralization of future emerging rabies variants and risk for selection of viral escape mutants (89). Indeed, it is poorly effective against rabies viruses carrying the N336D mutation in G protein, a variant identified in most of 60% of the African isolates, and which is not uncommon in North America (90).The combination, that targets antigenic sites I and III of the G protein, may overcome these limitations (91). Given together, these mAbs have been found able to neutralize multiple rabies virus lineages and to protect Syrian hamsters from a lethal rabies virus challenge (92). However, when compared to traditional human rabies immunoglobulin, the two preparations had similar prophylactic effect (93).

## 3 Examples of monoclonal antibodies against bacteria

With the evidence that antimicrobial resistance to commonly used antibiotics was progressively increasing and the development of new antibiotics effective on resistant pathogens was not fast enough to cover therapeutic needs, interest to mABs capable of inhibiting bacterial infections by neutralizing bacterial toxins and killing pathogenic bacteria has significantly raised (94). Several antibacterial antibodies, mainly targeting *Bacillus anthracis (Ba*), *Clostridium difficile* (*Cd*), *Staphylococcus aureus* (*Sa*) and *Pseudomonas aeruginosa* (*Pa*) have been developed and tested. Only 3 are authorized for use in humans to date: raxibacumab, obiltoxaximab, and bezlotoxumab (94).

Raxibacumab is a human mAb that prevents anthrax toxin-mediated cell damage through the inactivation of a component of the anthrax toxin (protective antigen) and the following inhibition of the lethal toxin internalization (95). It has been found effective in rabbits and monkeys and is approved for the intravenous treatment of adults and children with inhalational anthrax in combination with appropriate antibacterial drugs, and for prophylaxis of inhalational anthrax when alternative therapies are not available or are not appropriate (96, 97). Efficacy was established in experimental animals, whereas safety and dosages have been evaluated in adult volunteers (98). Pediatric dosages were extrapolated from adult pharmacokinetics (99). Obiltoxaximab is a second mAB potentially effective against *Ba* (100). It targets the same anthrax toxin component as raxibacumab, is given intravenously, and is authorized for the same indications as raxibacumab.

Bezlotoxumab is a mAB potentially effective against *Cd* (101). It neutralizes toxin B, the most potent toxin of this pathogen. It is supposed that this mAB acts when bacterial toxins alter the epithelial cells and disrupt the gut wall barrier function. This allows paracellular translocation of the mAB to the intestinal lumen, followed by neutralization of the toxin, recovery of the epithelium, and reestablishment of gut barrier (101). After evidence of a positive effect in experimental animals, bezlotoxumab was evaluated in humans. Phase 3 trials have shown that intravenous infusion of antibody bezlotoxumab as adjunct treatment for *Cd* infection significantly reduces the risk of recurrences over 12 weeks for adult patients with identified risk factors for recurrence (age >65 years, prior history of *Cd* infections, immunocompromised, and *Cd* infection severity). Patients without risk factors had no benefit (101). Starting from these premises, this mAB is approved to reduce recurrence of *Cd* infection in patients 18 years of age or older who are receiving antibacterial drug treatment of *Cd* infection and are at high risk for recurrence. Children are excluded. Bezlotoxumab should only be used in conjunction with antibacterial drug. Moreover, although generally safe, heart failure can follow administration and its use in patients with previously diagnosed heart problems should receive this mAB only when the advantages overcome the potential risk (102).

Regarding *Sa*, several mABs targeting bacterial antigens with relevant importance in the pathogenesis of *Sa* infection have been developed in recent years although none of them has been licensed for human use (102). Among them, are those directed against adhesins, cell-wall modifying enzymes, surface glycopolymers, biofilm matrix components, and toxins (103). Results of initial studies were negative for some of them, and this led to their exclusion from further development. Monoclonal ABs targeting alpha-hemolysin (α-HL), a key virulence factor of *Sa*, got the most attention. This factor can damage red blood cells, promote ischemic necrosis and induce cell apoptosis (104, 105). The greatest part of circulating *Sa*, including methicillin-resistant *Sa* (MRSA), possess this toxin and this explains why its inactivation was considered a potential measure to control difficult to treat *Sa* infections. The evidence that pathogenicity of bacteria with increased α-HL expression was significantly higher than that of normal pathogens (106) and that mutations of α-HL were associated with lower disease severity (107) strongly supported this conclusion. Unfortunately, as in animals, results of human studies were conflicting and this explains why none of them is presently on the market, despite some of them continue to be evaluated in clinical trials. KBSA301 was found effective in improving *Sa* eradication in hospitalized patients with severe pneumonia requiring admission to intensive care unit. In contrast, suvratoxumab failed in preventing *Sa* pneumonia in patients given mechanical ventilation (108). To improve mAB efficacy against *Sa*, a combination of two co-administered fully human mABs, ASN-1 and ASN-2, in a preparation named ASN100 was developed. ASN-1 neutralizes α-HL and four leukocidins whereas ASN-2 neutralizes a fifth leukocidin. The combination showed detectable penetration in the epithelial lining fluid (109). Use in mechanically ventilated patients was disappointing as the combination failed to prove its effectiveness in high-risk, mechanically ventilated patients with *Sa* pneumonia leading to the end of the mAB development (110).

Starting from the evidence that in most patients with chronic lung disease the emergence of resistance to antibiotics is a major obstacle to effective control of *Pa* infections, mABs with potential activity against this pathogen were developed. Studies have led to the production of mABs targeting epitopes *of Pa* PSL, an exosaccharide required for biofilm formation that also reduces host phagocytic function (111), and the PcrV protein, which is a critical needle tip protein of the type III secretion system of *Pa* so favoring cytotoxicity by bacterial toxin injection into host cell cytoplasm, bypassing the extracellular milieu (112). Compared to patients receiving standard treatment, adjunctive immunotherapy with panomacumab, a mAB directed against PSL, was associated with a better clinical outcome of confirmed *Pa* 011 pneumonia, with a resolution rate of 85% versus 64% (P=0.048). and a shorter time to clinical resolution (8.0 versus 18.5; P=0.004) (113). However, as this mAB can inactivate only a part of O antigen *Pa* serotypes (114), it was feared that it could provide insufficient strain coverage.

KB001-A is an anti-PcrV PEGylated monoclonal antibody that was initially found effective in experimental animal studies in controlling *Pa* infection. Therefore, it was considered a promising nonantibiotic strategy to reduce airway inflammation and damage in *Pa* pneumonia in humans (115). However, as in a further study carried out in cystic fibrosis patients the administration of this mAB was associated with a marginal reduction of the *Pa* titer in sputum and with a very poor increase in lung function, KB001-A was not further developed (116). To overcome limitations of these mABs and increase strain and disease coverage, a bispecific mAB targeting both PSL and PcrV, MEDI3902 simultaneously, was developed. Unfortunately, despite MEDI3902 prophylaxis or treatment was protective in rabbit bloodstream and lung infection models, use in humans was poorly satisfactory (117). Although theoretically effective serum concentrations were demonstrated, primary efficacy endpoint of reduction in *Pa* pneumonia development in mechanically ventilated patients was achieved only in subjects with lower levels of baseline inflammatory markers, suggesting that MEDI 3902 could be used only a selected minority of *Pa* infected patients (118).

## 4 Conclusions

An ever-increasing number of mABs effective against viruses (**Table 1**) or bacteria (**Table 2**) have been developed, although differently for what has been observed in chronic diseases (i.e., Chron’s disease, rheumatoid arthritis, ankylosing spondylitis, multiple sclerosis, breast cancer, some forms of lymphoma and leukemia) only in a small number of cases they have been licensed and have reached the market for use in clinical practice. Although some examples of efficacy in therapy, additional to traditional antiviral or antibacterial therapy has been shown, their efficacy is generally significantly greater in prophylaxis. In addition, even if some viral infections for which mAbs are effective are associated with chronic diseases (i.e., RSV with asthma, SARS-CoV-2 with long COVID), their efficacy in therapy has been observed mainly in acute infections. Prophylaxis of infectious diseases is usually and most cost-effectively carried out with vaccines that in most cases are significantly more effective than mABs. When available, vaccines must be preferred over mABs. However, development of vaccines usually requires longer periods of time and for many important infectious agents no vaccines have been, or could be, developed. The example of RSV is illustrative in this regard. RSV infection in neonates and younger infants can be very dangerous. Vaccines for these subjects are not available. On the other hand, their efficacy, due to the immaturity of the immune system remains debatable. Maternal immunization is presently not possible, and protection offered by administration of vaccines to pregnant women generally confer protection only to a reduced number of children and for a shorter than desired period of time. The universal use of an effective, safe and easy to use mAB such as NSM could definitively solve one of the most important infectious problem of neonates and younger infants. The COVID-19 pandemic has shown that in emergency situations availability of mABs can significantly reduce the impact of emerging epidemics and pandemics: mABs were developed significantly earlier than vaccines and were shown to be effective when administered prophylactically or early after diagnosis to high-risk COVID-19 patients. Unfortunately, COVID-19 has clearly highlighted the most important limit of mABs, the possibility that they rapidly lose their effectiveness in the event of important genetic mutations of the infectious agent towards which they are directed. This means the need for a continuous monitoring of the efficacy of these preparations and their updating in case of variant emergence. Finally, despite development of effective mABs against bacteria is more complicated than those against viruses, mAB can play a role in treatment of multidrug-resistant bacterial infections.

**Table 1:** Summary of main monoclonal antibodies for prevention and therapy of viral infections.

	Action	Effect/ Efficacy	Indications	Dose	Half-Life	Cost	Potential Use
RSV prevention							
Palivizumab (23–36)	Humanized mAB Target RSV F protein, site II	45-82% against RSV related hospitalizations in high risk infants	Infants <29 weeks of gestational age; chronic lung disease of prematurity; hemodynamically significant congenital heart defect; selected cases of pulmonary abnormalities, neuromuscular disorders and severe immunodeficiency	15 mg/kg once a month (max 5) during RSV season	Short (17-26 days)	Very high	Reduced (high risk groups only)
Nirsevimab (40–51)	Humanized mAB Target RSV prefusion F protein, site ϕ	Preterms: 70,1% reduction on RSV-associated MALRTIs, 2,6% vs 9,5% on RSV disease compared to placebo and 78,4% reduction on hospitalization rate due to RSV-associated MALRTIs. Preterm and Term Infants: 74,5% reduction on RSV-associated MALRTIs and 62,1% reduction on hospitalization	Universal indication (potential) on infants	Single dose: 50 mg IM in <5 kg and 100 mg IM in > 5kg	Extended half-life due to modified Fc region (≈150 days)	Potential favorable cost-effectiveness ratio	Universal use of RSV prophylaxis in children
Clesrovimab (52)	Humanized mAB Target RSV prefusion F protein, site IV	Studies ongoing					
SARS-CoV-2 prophylaxis or therapy							
Bebtelovimab (66–73)	Human IgG1 mAB Target SARS-CoV-2 S-protein. Active against Omicron BA.5 and Omicron BA.4.6/BF.7	Reduction on viral load on day 5 after treatment, compared to placebo. Time to sustained symptom resolution was reduced by a median of 2 days	Emergency use in adults and children >12 years of age and weighting >40 kg. Not authorized in hospitalized patients	175 mg IV	N/A	High	Patients with mild to moderate disease at high risk to progression.
Tixagevimab plus cilgavimab (78, 79)	Active against Omicron BA.5.	*In vivo* efficacy clearly evidenced only in patients infected by non-Omicron variants. i	Emergency use in adults and children >12 years of age and weighting >40 kg for pre-exposure prophylaxis in immunocompromised individuals or those who cannot be vaccinated or mount post-vaccination immune response	300 mg + 300 mg IM	N/A	Hugh	Post-exposition prophylaxis in selected patients with infection due to sensitive SARS-CoV-2 strains
HIV therapy							
Ibalizumab (81–83)	Humanized mAB Target CD4 T cell extracellular domain 1 and 2	*In vitro* neutralizing activity against approximately 90% of a diverse panel of HIV strains. Very fast reduction of HIV-RNA levels. After 24 weeks of treatment 43% of participants achieve HIV RNA suppression	In adults as part of a combination antiretroviral regimen in heavily treatment-experienced patients with multidrug resistant (MDR) HIV-1 infection who did not respond to the current antiretroviral regimen	First dose: 2000 mg IV; maintenance doses 800 mg every 2 weeks	Extended half-life with high dose due to saturable elimination	Very high	Only in patients unresponsive to antivirals
Rabies prevention							
Rabishield	Target a conformational epitope of the rabies G protein	It is as effective as serum derived hyperimmune IgG. It can fail against virus variants tat circulate in Africa and North America	It must be given together with vaccine within 7 days after exposure to rabies virus	3.33 IU/kg body weight		Less expensive than hyperimmune rabies IgG	Less recommended than preparations with two mABs
Miromavimab plus Docaravimab	Target the antigenic sites I and III of the rabies G protein	Combination is as effective as serum derived hyperimmune IgG	It must be given together with vaccine within 7 days after exposure to rabies virus	40 IU/kg body weight		Less expensive than hyperimmune rabies IgG	Extensive use

N/A, not applicable.

**Table 2:** Summary of main monoclonal antibodies for therapy of bacterial infections.

	Action	Effect/ Efficacy	Indications
Raxibacumab Obiltoxaximab (84–88)	Human mAB Inactivation of Anthrax toxin. Inhibition of lethal toxin internalization	In experimental animal models	IV treatment of adults and children with inhalational anthrax and for prophylaxis of inhalational anthrax
Bezlotoxumab (89, 90)	Human mAB Neutralize toxin B of *Clostridium difficile*.	Reduces the risk of recurrence over 12 weeks for adults patients with risk factors (aged >65 years, prior history of Cd infections, immunocompromised, Cd infection severity)	Approved to reduce recurrence of Cd infection in patients >18 years of age who are receiving antibacterial drug treatment and are at high risk for recurrence

## Author contributions

SE proposed the project, wrote the first draft of the manuscript and coordinated the study group. GA gave a significant contribution on viral infections. MB gave a significant contribution on SARS-CoV-2, HIV and bacterial infections. FB gave a significant contribution on RSV, SARS-CoV-2, *Sa* and *Pa* infections. FR gave a significant contribution on SARS-CoV-2, HIV and bacterial infections, NH gave a significant contribution on RSV, SARS-CoV-2 and *Sa* infections, IH gave a significant contribution on SARS-CoV-2, HIV and bacterial infections, AO reviewed and edited the whole manuscript, provided comments, suggested references and substantially contributed to the content of the manuscript. TT gave a significant contribution on RSV, SARS-CoV-2 and *Sa* infections. JT gave a significant contribution on RSV, SARS-CoV-2, *Sa* and *Pa* infections. AV gave a significant contribution on SARS-CoV-2, HIV and bacterial infections. NP co-wrote the first draft of the manuscript. All authors contributed to the article and approved the submitted version.

## References

[1] Raju TN. Emil Adolf Von behring and serum therapy for diphtheria. Acta Paediatr (2006) 95:258–9. doi: 10.1111/j.1651-2227.2006.tb02222.x 16497632

[2] João C Negi VS Kazatchkine MD Bayry J Kaveri SV. Passive serum therapy to immunomodulation by IVIG: a fascinating journey of antibodies. J Immunol (2018) 200:1957–63. doi: 10.4049/jimmunol.1701271 29507120

[3] Oncley JL Melin M Richert DA Cameron JW Gross PM. The separation of the antibodies, isoagglutinins, prothrombin, plasminogen and beta1-lipoprotein into subfractions of human plasma. J Am Chem Soc (1949) 71:541–50. doi: 10.1021/ja01170a048 18112064

[4] Marasco W Sui J. The growth and potential of human antiviral monoclonal antibody therapeutics. Nat Biotechnol (2007) 25:1421–34. doi: 10.1038/nbt1363 PMC7097443 18066039

[5] Köhler G Milstein C. Continuous cultures of fused cells secreting antibody of predefined specificity. Nature (1974) 256:495–7. doi: 10.1038/256495a0 1172191

[6] Zahavi D Weiner L. Monoclonal antibodies in cancer therapy. Antibodies (Basel) (2020) 9:34. doi: 10.3390/antib9030034 32698317 PMC7551545

[7] Pantaleo G Correia B Fenwick C Joo VS Perez L. Antibodies to combat viral infections: development strategies and progress. Nat Rev Drug Discovery (2022) 21:676–96. doi: 10.1038/s41573-022-00495-3 PMC9207876 35725925

[8] Otsubo R Yasui T. Monoclonal antibody therapeutics for infectious diseases: beyond normal human immunoglobulin. Pharmacol Ther (2022) 240:108233. doi: 10.1016/j.pharmthera.2022.108233 35738431 PMC9212443

[9] Walker LM Burton DR. Passive immunotherapy of viral infections: ‘super-antibodies’ enter the fray. Nat Rev Immunol (2018) 18:297–308. doi: 10.1038/nri.2017.148 29379211 PMC5918154

[10] Marston HD Paules CI Fauci AS. Monoclonal antibodies for emerging infectious diseases - borrowing from history. N Engl J Med (2018) 378:1469–72. doi: 10.1056/NEJMp1802256 29513615

[11] Bianchini S Silvestri E Argentiero A Fainardi V Pisi G Esposito S. Role of respiratory syncytial virus in pediatric pneumonia. Microorganisms (2020) 8:2048. doi: 10.3390/microorganisms8122048 33371276 PMC7766387

[12] Díez-Domingo J Pérez-Yarza EG Melero JA Sánchez-Luna M Aguilar MD Blasco AJ. Social, economic, and health impact of the respiratory syncytial virus: a systematic search. BMC Infect Dis (2014) 14:544. doi: 10.1186/s12879-014-0544-x 25358423 PMC4219051

[13] Herring WL Zhang Y Shinde V Stoddard J Talbird SE Rosen B. Clinical and economic outcomes associated with respiratory syncytial virus vaccination in older adults in the united states. Vaccine (2022) 40:483–93. doi: 10.1016/j.vaccine.2021.12.002 34933763

[14] Esposito S Piralla A Zampiero A Bianchini S Di Pietro G Scala A. Characteristics and their clinical relevance of respiratory syncytial virus types and genotypes circulating in northern Italy in five consecutive winter seasons. PloS One (2015) 10:e0129369. doi: 10.1371/journal.pone.0129369 26047100 PMC4457818

[15] Centers for Disease Control and Prevention. Respiratory syncytial virus (RSV) surveillance. Available at: https://www.cdc.gov/surveillance/nrevss/rsv/index.html (Accessed October 16, 2022).

[16] Bardsley M Morbey RA Hughes HE Beck CR Watson CH Zhao H. Epidemiology of respiratory syncytial virus in children younger than 5 years in England during the COVID-19 pandemic, measured by laboratory, clinical, and syndromic surveillance: a retrospective observational study. Lancet Infect Dis (2023) 23:56–66. S1473-3099(22)00525-4. 10.1016/S1473-3099(22)00525-4 PMC9762748 36063828

[17] Tempia S Walaza S Bhiman JN McMorrow ML Moyes J Mkhencele T. Decline of influenza and respiratory syncytial virus detection in facility-based surveillance during the COVID-19 pandemic, south Africa, January to October 2020. Euro Surveill (2021) 26sss:2001600. doi: 10.2807/1560-7917.ES.2021.26.29.2001600 PMC8299743 34296675

[18] Centers for Disease Control and Prevention. Changes in influenza and other respiratory virus activity during the COVID-19 pandemic Vol. 70. United States, 2020–2021: MMWR (2021) p. 1013–9. 10.15585/mmwr.mm7029a1 PMC8297694 34292924

[19] Kim HW Canchola JG Brandt CD Pyles G Chanock RM Jensen K. Respiratory syncytial virus disease in infants despite prior administration of antigenic inactivated vaccine. Am J Epidemiol (1969) 89:422–34. doi: 10.1093/oxfordjournals.aje.a120955 4305198

[20] Esposito S Di Pietro G. Respiratory syncytial virus vaccines: an update on those in the immediate pipeline. Future Microbiol (2016) 11:1479–90. doi: 10.2217/fmb-2016-0106 27750448

[21] Abbasi J. RSV Vaccines, finally within reach, could prevent tens of thousands of yearly deaths. JAMA (2022) 327:204–6. doi: 10.1001/jama.2021.23772 34964806

[22] Villanueva DH Arcega V Rao M. Review of respiratory syncytial virus infection among older adults and transplant recipients. Ther Adv Infect Dis (2022) 9:20499361221091413. doi: 10.1177/20499361221091413 35464624 PMC9019318

[23] The IMpact-RSV Study Group. Palivizumab, a humanized respiratory syncytial virus monoclonal antibody, reduces hospitalization from respiratory syncytial virus infection in high-risk infants. Pediatrics (1998) 102:531–7. doi: 10.1542/peds.102.3.531 9724660

[24] Feltes TF Cabalka AK Meissner HC Piazza FM Carlin DA Top FH Jr. Palivizumab prophylaxis reduces hospitalization due to respiratory syncytial virus in young children with hemodynamically significant congenital heart disease. J Pediatr (2003) 143:532–40. doi: 10.1067/S0022-3476(03)00454-2 14571236

[25] Blanken MO Rovers MM Molenaar JM Winkler-Seinstra PL Meijer A Kimpen JL. Respiratory syncytial virus and recurrent wheeze in healthy preterm infants. N Engl J Med (2013) 368:1791–9. doi: 10.1056/NEJMoa1211917 23656644

[26] American Academy of Pediatrics Committee on Infectious Diseases and Committee of Fetus and Newborn. Prevention of respiratory syncytial virus infections: indications for the use of palivizumab and update on the use of RSV-IGIV. Pediatrics (1998) 102:1211–6. doi: 10.1542/peds.102.5.1211 9794957

[27] Cassel CK Guest JA. Choosing wisely: helping physicians and patients make smart decisions about their care. JAMA (2012) 307:1801–2. doi: 10.1001/jama.2012.476 22492759

[28] ABIM Foundation. American Board of Internal Medicine ACP-ASIM Foundation. American College of Physicians-American Society of Internal Medicine European Federation of Internal Medicine. Medical professionalism in the new millennium: a physician charter. Ann Intern Med (2002) 136:243–6. doi: 10.7326/0003-4819-136-3-200202050-00012 11827500

[29] Ubel PA Jagsi R. Promoting population health through financial stewardship. N Engl J Med (2014) 370:1280–1. doi: 10.1056/NEJMp1401335 24693887

[30] Zhu Q Patel NK McAuliffe JM Zhu W Wachter L McCarthy MP. Natural polymorphisms and resistance-associated mutations in the fusion protein of respiratory syncytial virus (RSV): effects on RSV susceptibility to palivizumab. J Infect Dis (2012) 205:635–8. doi: 10.1093/infdis/jir790 22184728

[31] American Academy of Pediatrics Committee on Infectious Diseases American Academy of Pediatrics Bronchiolitis Guidelines Committee. Updated guidance for palivizumab prophylaxis among infants and young children at increased risk of hospitalization for respiratory syncytial virus infection. Pediatrics (2014) 134:415–20. doi: 10.1542/peds.2014-2783 25070315

[32] Sáez-Llorens X Moreno MT Ramilo O Sánchez PJ Top FH Jr Connor EM. Safety and pharmacokinetics of palivizumab therapy in children hospitalized with respiratory syncytial virus infection. Pediatr Infect Dis J (2004) 23:707–17. doi: 10.1097/01.inf.0000133165.85909.08 15295219

[33] Subramanian KN Weisman LE Rhodes T Ariagno R Sánchez PJ Steichen J. Safety, tolerance and pharmacokinetics of a humanized monoclonal antibody to respiratory syncytial virus in premature infants and infants with bronchopulmonary dysplasia. MEDI-493 Study Group Pediatr Infect Dis J (1998) 17:110–5. doi: 10.1097/00006454-199802000-00006 9493805

[34] Johnson S Oliver C Prince GA Hemming VG Pfarr DS Wang SC. Development of a humanized monoclonal antibody (MEDI-493) with potent *in vitro* and *in vivo* activity against respiratory syncytial virus. J Infect Dis (1997) 176:1215–24. doi: 10.1086/514115 9359721

[35] Frogel MP Stewart DL Hoopes M Fernandes AW Mahadevia PJ. A systematic review of compliance with palivizumab administration for RSV immunoprophylaxis. J Manag Care Pharm (2010) 16:46–58. doi: 10.18553/jmcp.2010.16.1.46 20131495 PMC10437841

[36] Mac S Sumner A Duchesne-Belanger S Stirling R Tunis M Sander B. Cost-effectiveness of palivizumab for respiratory syncytial virus: a systematic review. Pediatrics (2019) 143:e20184064. doi: 10.1542/peds.2018-4064 31040196

[37] Jones RG Martino A. Targeted localized use of therapeutic antibodies: a review of non-systemic, topical and oral applications. Crit Rev Biotechnol (2016) 36:506–20. 10.3109/07388551.2014.992388 25600465

[38] Export this cochrane central register of controlled trials. nasal administration of palivizumab to prevent respiratory syncytial virus infection (2018). Available at: https://trialsearch-who-int.pros1.lib.unimi.it/Trial2.aspx?TrialID=EUCTR2018-002742-37-NL (Accessed October 10, 2022).

[39] Adis insight. Palivizumab biosimilar - mAbxience. Available at: https://adisinsight.springer.com/drugs/800046954 (Accessed October 16, 2022).

[40] McLellan JS. Neutralizing epitopes on the respiratory syncytial virus fusion glycoprotein. Curr Opin Virol (2015) 11:70–5. doi: 10.1016/j.coviro.2015.03.002 PMC4456247 25819327

[41] Dall’Acqua WF Kiener PA Wu H. Properties of human IgG1s engineered for enhanced binding to the neonatal fc receptor (FcRn). J Biol Chem (2006) 281:23514–24. doi: 10.1074/jbc.M604292200 16793771

[42] Esposito S Abu-Raya B Bonanni P Cahn-Sellem F Flanagan KL Martinon Torres F. Coadministration of anti-viral monoclonal antibodies with routine pediatric vaccines and implications for nirsevimab use: a white paper. Front Immunol (2021) 12:708939. doi: 10.3389/fimmu.2021.708939 34456918 PMC8386277

[43] European Medicines Agency. New medicine to protect babies and infants from respiratory syncytial virus (RSV) infection. Available at: https://www.ema.europa.eu/en/news/new-medicine-protect-babies-infants-respiratory-syncytial-virus-rsv-infection (Accessed October 16, 2022).

[44] Domachowske JB Khan AA Esser MT Jensen K Takas T Villafana T. Safety, tolerability and pharmacokinetics of MEDI8897, an extended half-life single-dose respiratory syncytial virus prefusion f-targeting monoclonal antibody administered as a single dose to healthy preterm infants. Pediatr Infect Dis J (2018) 37:886–92. doi: 10.1097/INF.0000000000001916 PMC6133204 29373476

[45] Hammitt LL Dagan R Yuan Y Baca Cots M Bosheva M Madhi. Nirsevimab for prevention of RSV in healthy late-preterm and term infants. N Engl J Med (2022) 386:837–46. doi: 10.1056/NEJMoa2110275 35235726

[46] Zhu Q McLellan JS Kallewaard NL Ulbrandt ND Palaszynski S Zhang J. A highly potent extended half-life antibody as a potential RSV vaccine surrogate for all infants. Sci Transl Med (2017) 9:eaaj1928–eaaj1928. doi: 10.1126/scitranslmed.aaj1928 28469033

[47] Esposito S Abu Raya B Baraldi E Flanagan K Martinon Torres F Tsolia M. RSV Prevention in all infants: which is the most preferable strategy? Front Immunol (2022) 13:880368. doi: 10.3389/fimmu.2022.880368 35572550 PMC9096079

[48] Bont L Weil Olivier C Herting E Esposito S Navarro Alonso JA Lega F. The assessment of future RSV immunizations: how to protect all infants? Front Pediatr (2022) 10:981741. doi: 10.3389/fped.2022.981741 36016878 PMC9396232

[49] Simões EAF Madhi SA Muller WJ Atanasova V Bosheva M Cabañas F. Efficacy of nirsevimab against respiratory syncytial virus lower respiratory tract infections in preterm and term infants, and pharmacokinetic extrapolation to infants with congenital heart disease and chronic lung disease: a pooled analysis of randomised controlled trials. Lancet Child Adolesc Health (2023) 7:180–9. doi: 10.1016/S2352-4642(22)00321-2 PMC9940918 36634694

[50] Kieffer A Beuvelet M Sardesai A Musci R Milev S Roiz J. Expected impact of universal immunization with nirsevimab against RSV-related outcomes and costs among all US infants in their first RSV season: a static model. J Infect Dis (2022) 226(Suppl 2):S282–92. doi: 10.1093/infdis/jiac216 PMC9377043 35968866

[51] Voirin N Virlogeux V Demont C Kieffer A. Potential impact of nirsevimab on RSV transmission and medically attended lower respiratory tract illness caused by RSV: a disease transmission model. Infect Dis Ther (2022) 11:277–92. doi: 10.1007/s40121-021-00566-9 PMC8847469 34813073

[52] Merck. Clesrovimab in infants and children at increased risk for severe respiratory syncytial virus disease. Available at: https://www.merckclinicaltrials.com/trial/nct04938830/ (Accessed 30 January, 2023).

[53] World health Organization. WHO coronavirs (COVID-19) dashboard. Available at: https://covid19.who.int/ (Accessed March 25, 2023).

[54] Luo T Cao Z Wang Y Zeng D Zhang Q. Role of asymptomatic COVID-19 cases in viral transmission: findings from a hierarchical community contact network model. IEEE Trans Autom Sci Eng (2021) 19:576–85. doi: 10.1109/TASE.2021.3106782 PMC9088818 35582345

[55] World Health Organization. Impact of COVID-19 on people’s livelihoods, their health and our food systems. Available at: https://www.who.int/news/item/13-10-2020-impact-of-covid-19-on-people’s-livelihoods-their-health-and-our-food-systems#:~:text=The%20economic%20and%20social%20disruption,the%20end%20of%20the%20year (Accessed October 25, 2022).

[56] Centers for Disease Control and Prevention. Risk for COVID-19 infection, hospitalization, and death by age group. Available at: https://www.cdc.gov/coronavirus/2019-ncov/covid-data/investigations-discovery/hospitalization-death-by-age.html (Accessed October 25, 2022).

[57] Esposito S Autore G Argentiero A Ramundo G Perrone S Principi N. Update on COVID-19 therapy in pediatric age. Pharm (Basel) (2022) 15:1512. doi: 10.3390/ph15121512 PMC9783267 36558963

[58] American Academy of Pediatrics and the Children’s Hospital Association. Children and COVID-19: state data report. Available at: https://downloads.aap.org/AAP/PDF/AAP%20and%20CHA%20-%20Children%20and%20COVID-19%20State%20Data%20Report%209.29.22%20FINAL.pdf?_ga=2.170767266.309218142.1664893746-995825483.1664893746 (Accessed October 6, 2022).

[59] Esposito S Principi N. Multisystem inflammatory syndrome in children related to SARS-CoV-2. Paediatr Drugs (2021) 23:119–29. doi: 10.1007/s40272-020-00435-x PMC7819738 33479801

[60] More K Aiyer S Goti A Parikh M Sheikh S Patel G. Multisystem inflammatory syndrome in neonates (MIS-n) associated with SARS-CoV2 infection: a case series. Eur J Pediatr (2022) 181:1883–98. doi: 10.1007/s00431-022-04377-z PMC8759431 35031848

[61] Gurdasani D Akrami A Bradley VC Costello A Greenhalgh T Flaxman S. Long COVID in children. Lancet Child Adolesc Health (2022) 6:e2. doi: 10.1016/S2352-4642(21)00342-4 34921807 PMC8673872

[62] Esposito S Principi N Azzari C Cardinale F Di Mauro G Galli L. Italian Intersociety consensus on management of long covid in children. Ital J Pediatr (2022) 48:42. doi: 10.1186/s13052-022-01233-6 35264214 PMC8905554

[63] Fainardi V Meoli A Chiopris G Motta M Skenderaj K Grandinetti R. Long COVID in children and adolescents. Life (Basel) (2022) 12:285. doi: 10.3390/life12020285 35207572 PMC8876679

[64] Walls AC Park YJ Tortorici MA Wall A McGuire AT Veesler D. Structure, function, and antigenicity of the SARS-CoV-2 spike glycoprotein. Cell (2020) 181:281–292.e6. doi: 10.1016/j.cell.2020.02.058 32155444 PMC7102599

[65] Kelley B. Developing therapeutic monoclonal antibodies at pandemic pace. Nat Biotechnol (2020) 38:540–5. doi: 10.1038/s41587-020-0512-5 PMC7173776 32317764

[66] Hwang YC Lu RM Su SC Chiang PY Ko SH Ke FY. Monoclonal antibodies for COVID-19 therapy and SARS-CoV-2 detection. J BioMed Sci (2022) 29:1. doi: 10.1186/s12929-021-00784-w 34983527 PMC8724751

[67] Kreuzberger N Hirsch C Chai KL Tomlinson E Khosravi Z Popp M. SARS-CoV-2-neutralising monoclonal antibodies for treatment of COVID-19. Cochrane Database Syst Rev (2021) 9:CD013825. 34473343 10.1002/14651858.CD013825.pub2 PMC8411904

[68] Bahakel H Murphy C Frenck RW Jr Grimley MS Marsh RA Paulsen GC. Single site experience of the use of monoclonal antibodies for the treatment of COVID-19 in high-risk pediatric and young adult patients. Pediatr Infect Dis J (2022) 41:985–8. doi: 10.1097/INF.0000000000003703 PMC9645449 36219876

[69] Lanari M Venturini E Pierantoni L Stera G Castelli Gattinara G Esposito SMR. Eligibility criteria for pediatric patients who may benefit from anti SARS-CoV-2 monoclonal antibody therapy administration: an Italian inter-society consensus statement. Ital J Pediatr (2022) 48:7. doi: 10.1186/s13052-021-01187-1 35022088 PMC8754075

[70] Cao Y Yisimayi A Jian F Song W Xiao T Wang L. BA.2.12.1, BA.4 and BA.5 escape antibodies elicited by omicron infection. Nature (2022) 608:593–602. doi: 10.1038/s41586-022-04980-y 35714668 PMC9385493

[71] Wang Q Guo Y Iketani S Nair MS Li Z Mohri H. Antibody evasion by SARS-CoV-2 omicron subvariants BA.2.12.1, BA.4, and BA.5. Nature (2022) 608:603–8. doi: 10.1038/s41586-022-05053-w PMC9385487 35790190

[72] Yamasoba D Kosugi Y Kimura I Fujita S Uriu K Ito J. Neutralisation sensitivity of SARS-CoV-2 omicron subvariants to therapeutic monoclonal antibodies. Lancet Infect Dis (2022) 22:942–3. doi: 10.1016/S1473-3099(22)00365-6 PMC9179126 35690075

[73] Case JB Mackin S Errico JM Chong Z Madden EA Whitener B. Resilience of S309 and AZD7442 monoclonal antibody treatments against infection by SARS-CoV-2 omicron lineage strains. bioRxiv (2022) 2022:3. doi: 10.1101/2022.03.17.4847874 PMC9250508 35780162

[74] US Food and Drug Administration. Fact sheet for healthcare providers: emergency use authorization for evusheldtm (Tixagevimab Co-packaged with cilgavimab). Available at: https://www.fda.gov/media/154701/download https://www.ema.europa.eu/en/documents/product-information/evusheld-epar-product-information_en.pdf (Accessed October 20, 2022). European Medicines Agency EVUSHELD Product Information. Available at. Accessd on October 20, 2022.

[75] VanBlargan LA Errico JM Halfmann PJ Zost SJ Crowe JE Jr Purcell LA. An infectious SARS-CoV-2 B.1.1.529 omicron virus escapes neutralization by therapeutic monoclonal antibodies. Nat Med (2022) 28:490–5. doi: 10.1038/s41591-021-01678-y PMC8767531 35046573

[76] U.S. Food and Drug Administration. Coronavirus (COVID-19) update: FDA authorizes new monoclonal antibody for treatment of COVID-19 that retains activity against omicron variant. Available at: https://www.fda.gov/news-events/press-announcements/coronavirus-covid-19-update-fda-authorizes-new-monoclonal-antibody-treatment-covid-19-retain (Accessed October 20, 2022).

[77] Dougan M Azizad M Chen P Feldman B Frieman M Igbinadolor A. Bebtelovimab, alone or together with bamlanivimab and etesevimab, as a broadly neutralizing monoclonal antibody treatment for mild to moderate, ambulatory COVID-19. medRxiv (2022). doi: 10.1101/2022.03.10.22272100. 03.10.22272100.

[78] U.S. Food and Drug Administration. Coronavirus (COVID-19) update: FDA authorizes new long-acting monoclonal antibodies for pre-exposure prevention of COVID-19 in certain individuals. Available at: https://www.fda.gov/news-events/press-announcements/coronavirus-covid-19-update-fda-authorizes-new-long-acting-monoclonal-antibodies-pre-exposure (Accessed October 20, 2022).

[79] Levin MJ Ustianowski A De Wit S Launay O Avila M Templeton A. Intramuscular AZD7442 (Tixagevimab-cilgavimab) for prevention of covid-19. N Engl J Med (2022) 386:2188–200. doi: 10.1056/NEJMoa2116620 PMC9069994 35443106

[80] Kufel WD. Antibody-based strategies in HIV therapy. Int J Antimicrob Agents (2020) 56:106186. doi: 10.1016/j.ijantimicag.2020.106186 33045349 PMC7546180

[81] Beccari MV Mogle BT Sidman EF Mastro KA Asiago-Reddy E Kufel WD. Ibalizumab, a novel monoclonal antibody for the management of multidrug-resistant HIV-1 infection. Antimicrob Agents Chemother (2019) 63:e00110–19. doi: 10.1128/AAC.00110-19 PMC6535568 30885900

[82] U.S. Food and Drug Administration. FDA Approves new HIV treatment for patients who have limited treatment options. Available at: https://www.fda.gov/news-events/press-announcements/fda-approves-new-hiv-treatment-patients-who-have-limited-treatment-options (Accessed October 20, 2022).

[83] Blair HA. Ibalizumab: a review in multidrug-resistant HIV-1 infection. Drugs (2020) 80:189–96. doi: 10.1007/s40265-020-01258-3 31970712

[84] Hampson K Coudeville L Lembo T Sambo M Kieffer A Attlan M. Estimating the global burden of endemic canine rabies. PloS Negl Trop Dis (2015) 9:e0003709. 25881058 10.1371/journal.pntd.0003709 PMC4400070

[85] Kuzmina NA Kuzmin IV Ellison JA Rupprecht CE. Conservation of binding epitopes for monoclonal antibodies on the rabies virus glycoprotein. J Antivir Antiretrovir (2016) 5:37–43.

[86] World Health Organization. Meeting of the strategic advisory group of experts on immunization, October 2017 – conclusions and recommendations. Geneva, Switzerland: WHO (2017).

[87] Sparrow E Torvaldsen S Newall AT Wood JG Sheikh M Kieny MP. Recent advances in the development of monoclonal antibodies for rabies post exposure prophylaxis: a review of the current status of the clinical development pipeline. Vaccine (2019) 37 Suppl 1:A132–9. doi: 10.1016/j.vaccine.2018.11.004 30503659

[88] Fan L Zhang L Li J Zhu F. Advances in the progress of monoclonal antibodies for rabies. Hum Vaccin Immunother (2022) 18:2026713. doi: 10.1080/21645515.2022.2026713 35172707 PMC8993100

[89] Müller T Dietzschold B Ertl H Fooks AR Freuling C Fehlner-Gardiner C. Development of a mouse monoclonal antibody cocktail for post-exposure rabies prophylaxis in humans. PloS Negl Trop Dis (2009) 3:e542. 19888334 10.1371/journal.pntd.0000542 PMC2765635

[90] Wang W Ma J Nie J Li J Cao S Wang L. Antigenic variations of recent street rabies virus. Emerg Microbes Infect (2019) 8:1584–92. doi: 10.1080/22221751.2019.1683436 PMC6844422 31682199

[91] Gogtay NJ Munshi R Ashwath Narayana DH Mahendra BJ Kshirsagar V Gunale B. Comparison of a novel human rabies monoclonal antibody to human rabies immunoglobulin for postexposure prophylaxis: a phase 2/3, randomized, single-blind, noninferiority, controlled study. Clin Infect Dis (2018) 66:387–95. doi: 10.1093/cid/cix791 29020321

[92] De Benedictis P Minola A Rota Nodari E Aiello R Zecchin B Salomoni A. Development of broad-spectrum human monoclonal antibodies for rabies post-exposure prophylaxis. EMBO Mol Med (2016) 8:407–21. doi: 10.15252/emmm.201505986 PMC4818751 26992832

[93] Kansagra K Parmar D Mendiratta SK Patel J Joshi S Sharma N. A phase 3, randomised, open-label, non-inferiority trial evaluating anti-rabies monoclonal antibody cocktail (Twinrab TM) against human rabies immunoglobulin (HRIG). Clin Infect Dis (2021) 73:e2722–8. doi: 10.1093/cid/ciaa779 32556113

[94] Wang H Chen D Lu H. Anti-bacterial monoclonal antibodies: next generation therapy against superbugs. Appl Microbiol Biotechnol (2022) 106:3957–72. doi: 10.1007/s00253-022-11989-w 35648146

[95] Mazumdar S. Raxibacumab. mAbs (2009) 1:531–8. doi: 10.4161/mabs.1.6.10195 PMC2791309 20068396

[96] Migone TS Subramanian GM Zhong J Healey LM Corey A Devalaraja M. Raxibacumab for the treatment of inhalational anthrax. N Engl J Med (2009) 361:135–44. doi: 10.1056/NEJMoa0810603 19587338

[97] Kummerfeldt CE. Raxibacumab: potential role in the treatment of inhalational anthrax. Infect Drug Resist (2014) 7:101–9. doi: 10.2147/IDR.S47305 PMC4011807 24812521

[98] Greig SL. Obiltoxaximab: first global approval. Drugs (2016) 76:823–30. doi: 10.1007/s40265-016-0577-0 27085536

[99] Reichert JM. Antibodies to watch in 2017. MAbs (2017) 9:167–81. doi: 10.1080/19420862.2016.1269580 PMC5297518 27960628

[100] Johnson S Gerding DN. Bezlotoxumab. Clin Infect Dis (2019) 68:699–704. doi: 10.1093/cid/ciy577 30020417

[101] Wilcox MH Gerding DN Poxton IR Kelly C Nathan R Birch T. Bezlotoxumab for prevention of recurrent clostridium difficile infection. N Engl J Med (2017) 376:305–17. doi: 10.1056/NEJMoa1602615 28121498

[102] U.S. Food and Drug Administration. Drug trials snapshots: ZINPLAVA. Available at: https://www.fda.gov/drugs/drug-approvals-and-databases/drug-trials-snapshots-zinplava (Accessed October 20, 2022).

[103] Raafat D Otto M Reppschläger K Iqbal J Holtfreter S. Fighting *Staphylococcus aureus* biofilms with monoclonal antibodies. Trends Microbiol (2019) 27:303–22. doi: 10.1016/j.tim.2018.12.009 PMC6420399 30665698

[104] Kebaier C Chamberland RR Allen IC Gao X Broglie PM Hall JD. *Staphylococcus aureus* alpha-hemolysin mediates virulence in a murine model of severe pneumonia through activation of the NLRP3 inflammasome. J Infect Dis (2012) 205:807–17. doi: 10.1093/infdis/jir846 PMC3274379 22279123

[105] Powers ME Becker RE Sailer A Turner JR Bubeck Wardenburg J. Synergistic action of *Staphylococcus aureus* alpha-toxin on platelets and myeloid lineage cells contributes to lethal sepsis. Cell Host Microbe (2015) 17:775–87. doi: 10.1016/j.chom.2015.05.011 PMC4642999 26067604

[106] Tkaczyk C Hamilton MM Datta V Yang XP Hilliard JJ Stephens GL. *Staphylococcus aureus* alpha toxin suppresses effective innate and adaptive immune responses in a murine dermonecrosis model. PloS One (2013) 8:e75103. doi: 10.1371/journal.pone.0075103 24098366 PMC3788755

[107] Diep BA Hilliard JJ Le VT Tkaczyk C Le HN Tran VG. Targeting alpha toxin to mitigate its lethal toxicity in ferret and rabbit models of *Staphylococcus aureus* necrotizing pneumonia. Antimicrob Agents Chemother (2017) 61:e02456–16. doi: 10.1128/AAC.02456-16 PMC5365647 28115346

[108] François B Jafri HS Chastre J Sánchez-García M Eggimann P Dequin PF. Efficacy and safety of suvratoxumab for prevention of *Staphylococcus aureus* ventilator-associated pneumonia (SAATELLITE): a multicentre, randomised, double-blind, placebo-controlled, parallel-group, phase 2 pilot trial. Lancet Infect Dis (2021) 21:1313–23. doi: 10.1016/S1473-3099(20)30995-6 33894131

[109] Magyarics Z Leslie F Bartko J Rouha H Luperchio S Schörgenhofer C. Randomized, double-blind, placebo-controlled, single-Ascending-Dose study of the penetration of a monoclonal antibody combination (ASN100) targeting *Staphylococcus aureus* cytotoxins in the lung epithelial lining fluid of healthy volunteers. Antimicrob Agents Chemother (2019) 63:e00350–19. doi: 10.1128/AAC.00350-19 PMC6658777 31138568

[110] Genetic Engineering and Biotechnology Mews. Arsanis halts phase II trial of pneumonia candidate ASN100. Available at: https://www.genengnews.com/topics/drug-discovery/arsanis-halts-phase-ii-trial-of-pneumonia-candidate-asn100/ (Accessed October 20, 2022).

[111] Colvin KM Irie Y Tart CS Urbano R Whitney JC Ryder C. The pel and psl polysaccharides provide *Pseudomonas aeruginosa* structural redundancy within the biofilm matrix. Environ Microbiol (2012) 14:1913–28. doi: 10.1111/j.1462-2920.2011.02657.x PMC3840794 22176658

[112] Hauser A. The type III secretion system of *Pseudomonas aeruginosa*: infection by injection. Nat Rev Microbiol (2009) 7:654–65. doi: 10.1038/nrmicro2199 PMC2766515 19680249

[113] Que YA Lazar H Wolff M François B Laterre PF Mercier E. Assessment of panobacumab as adjunctive immunotherapy for the treatment of nosocomial *Pseudomonas aeruginosa* pneumonia. Eur J Clin Microbiol Infect Dis (2014) 33:1861–7. doi: 10.1007/s10096-014-2156-1 24859907

[114] Lu Q Eggimann P Luyt CE Wolff M Tamm M François B. *Pseudomonas aeruginosa* serotypes in nosocomial pneumonia: prevalence and clinical outcomes. Crit Care (2014) 18:R17. doi: 10.1186/cc13697 24428878 PMC4057348

[115] Sawa T Ito E Nguyen VH Haight M. Anti-PcrV antibody strategies against virulent pseudomonas aeruginosa. Hum Vaccin Immunother (2014) 10:2843–52. doi: 10.4161/21645515.2014.971641 PMC5443083 25483637

[116] Fierce Biotech. KaloBios reports top-line data for phase 2 study of KB001-a to treat pseudomonas aeruginosa lung infections in cystic fibrosis patients. Available at: https://www.fiercebiotech.com/biotech/kalobios-reports-top-line-data-for-phase-2-study-of-kb001-a-to-treat-pseudomonas-aeruginosa (Accessed October 20, 2022).

[117] Le HN Tran VG Vu TTT Gras E Le VTM Pinheiro MG. Treatment efficacy of MEDI3902 in *Pseudomonas aeruginosa* bloodstream infection and acute pneumonia rabbit models. Antimicrob Agents Chemother (2019) 63:e00710–19. doi: 10.1128/AAC.00710-19 PMC6658784 31160288

[118] Chastre J François B Bourgeois M Komnos A Ferrer R Rahav G. Efficacy, pharmacokinetics (PK), and safety profile of MEDI3902, an anti-*Pseudomonas aeruginosa* bispecific human monoclonal antibody in mechanically ventilated intensive care unit patients; results of the phase 2 EVADE study conducted by the public-private COMBACTE-MAGNET consortium in the innovative medicines initiative (IMI) program, open forum. Infect Dis (2020) 7(Supplement 1):S377–8. doi: 10.1093/ofid/ofaa439.829

